# Search for new loci and low-frequency variants influencing glioma risk by exome-array analysis

**DOI:** 10.1038/ejhg.2015.170

**Published:** 2015-08-12

**Authors:** Ben Kinnersley, Yoichiro Kamatani, Marianne Labussière, Yufei Wang, Pilar Galan, Karima Mokhtari, Jean-Yves Delattre, Konstantinos Gousias, Johannes Schramm, Minouk J Schoemaker, Anthony Swerdlow, Sarah J Fleming, Stefan Herms, Stefanie Heilmann, Markus M Nöthen, Matthias Simon, Marc Sanson, Mark Lathrop, Richard S Houlston

**Affiliations:** 1 Division of Genetics and Epidemiology, The Institute of Cancer Research, Sutton, Surrey, UK; 2 Foundation Jean Dausset-CEPH, Paris, France; 3 Sorbonne Universités UPMC Univ Paris 06, INSERM CNRS, Paris, France; 4 Université Paris 13 Sorbonne Paris Cité, Inserm (U557), Cnam, Bobigny, France; 5 AP-HP, GH Pitié-Salpêtrière, Service de Neurologie Mazarin, Paris, France; 6 Groupe Hospitalier Pitié-Salpêtrière, Paris, France; 7 Department of Neurosurgery, University of Bonn Medical Center, Bonn, Germany; 8 Division of Breast Cancer Research, The Institute of Cancer Research, Sutton, Surrey, UK; 9 Centre for Epidemiology and Biostatistics, Faculty of Medicine and Health, University of Leeds, Leeds, UK; 10 Institute of Human Genetics, University of Bonn, Bonn, Germany; 11 Department of Biomedicine, Division of Medical Genetics, University of Basel, Basel, Switzerland; 12 Department of Human Genetics, Génome Québec, McGill University, Montreal, QC, Canada

## Abstract

To identify protein-altering variants (PAVs) for glioma, we analysed Illumina HumanExome BeadChip exome-array data on 1882 glioma cases and 8079 controls from three independent European populations. In addition to single-variant tests we incorporated information on the predicted functional consequences of PAVs and analysed sets of genes with a higher likelihood of having a role in glioma on the basis of the profile of somatic mutations documented by large-scale sequencing initiatives. Globally there was a strong relationship between effect size and PAVs predicted to be damaging (*P*=2.29 × 10^−49^); however, these variants which are most likely to impact on risk, are rare (MAF<5%). Although no single variant showed an association which was statistically significant at the genome-wide threshold a number represented promising associations – *BRCA2*:c.9976A>T, p.(Lys3326Ter), which has been shown to influence breast and lung cancer risk (odds ratio (OR)=2.3, *P*=4.00 × 10^−4^ for glioblastoma (GBM)) and *IDH2*:c.782G>A, p.(Arg261His) (OR=3.21, *P*=7.67 × 10^−3^, for non-GBM). Additionally, gene burden tests revealed a statistically significant association for *HARS2* and risk of GBM (*P*=2.20 × 10^−6^). Genome scans of low-frequency PAVs represent a complementary strategy to identify disease-causing variants compared with scans based on tagSNPs. Strategies to lessen the multiple testing burden by restricting analysis to PAVs with higher priors affords an opportunity to maximise study power.

## Introduction

Gliomas account for ~40% of all primary brain tumours and are diagnosed in around 26 000 individuals in Europe each year.^1, 2^ Gliomas are typically classified as being either glioblastoma (GBM) or non-GBM tumours (diffuse ‘low-grade' glioma WHO grade I/II and anaplastic glioma WHO grade III tumours).^3^ Most gliomas carry a poor prognosis, with the most common type, GBM, typically having a median survival of 15 months.^2^ The only environmental factor consistently shown to influence glioma risk is exposure to ionising radiation,^2^ which accounts for only a very small number of cases. Evidence for genetic predisposition to glioma is provided by rare inherited cancer syndromes including Turcot's and Li–Fraumeni syndromes, and neurofibromatosis.^2, 4^ Collectively however they account for little of the 2-fold increased risk of glioma seen in relatives of patients.^5^

Much of the variation in genetic risk of glioma appears to be polygenic. Support for this proposal has come from genome-wide association studies (GWAS) which have identified common single-nucleotide polymorphisms (SNPs) at six loci influencing risk – 5p15.33 (*TERT*), 7p11.2 (*EGFR*, two regions), 8q24.21 (*CCDC26*), 9p21.3 (*CDKN2A/CDKN2B*), 11q23.3 (*PHLDB1*) and 20q13.33 (*RTEL1*).^6, 7, 8^ Despite the success of GWAS such studies are not optimally configured to identify low-frequency variants with stronger effects. Protein altering variants (PAVs), which alter the encoded amino acid sequence, are proportionally less prevalent than synonymous variants; however, such variants are *a priori* more likely to have a functional impact. Coupled with the observation that Mendelian disease susceptibility is generally caused by coding sequence changes^9^ suggests that association studies formulated around a gene-centric approach may be a powerful strategy for identifying disease-causing associations.

Although no rare recurrent PAV has thus far been shown to influence glioma risk the low-frequency variants NM_007194.3(*CHEK2*):c.1100delC, p.(Thr367Metfs), NM_000059.3(*BRCA2)*:c.9976A>T, p.(Lys3326Ter) and NM_000038.5(*APC*):c.3920T>A, p.(Ile1307Lys) confer 2- to 3-fold risks of breast, lung and colorectal cancers (CRC) respectively.^10, 11, 12^ Additionally the observation that the NM_001128425.1(*MUTYH*):c.536A>G, p.(Tyr179Cys) and NM_001128425.1(*MUTYH*):c.1187G>A, p.(Gly396Asp) variants cause recessive polyposis and CRC^13^ provides a precedent for rare recurrent variants having substantive effects on cancer risk.

The advent of next generation sequencing is allowing the cataloguing of recurrent coding variation, making the search for disease-causing PAVs on a genome-wide basis a viable proposition. Here we have investigated the contribution of recurrent coding variants to glioma by analysing 1882 cases and 8079 controls genotyped using the Illumina HumanExome BeadChip. To increase our power to identify disease-causing variants, we jointly tested groups of variants in a gene and incorporated information on the predicted functional consequences of PAVs. In addition we restricted our analysis to sets of genes with a higher likelihood of having a role in glioma on the basis of somatic mutation profile.

## Materials and Methods

### Subjects

We analysed three non-overlapping case–control series of Northern European ancestry: the UK series comprised 605 glioma cases (63% male; mean age at diagnosis 46 years) ascertained through the INTERPHONE Study^14^ with 5964 individuals from the 1958 Birth Cohort (1958BC;^15^) with no known personal history of cancer serving as a controls; the French series comprised 906 incident cases of glioma ascertained through the Service de Neurologie Mazarin, Groupe Hospitalier Pitié-Salpêtrière, Paris^6^ and 699 controls from the SU.VI.MAX (SUpplementation en VItamines et MinerauxAntioXydants) study of 12 735 healthy subjects (women aged 35–60 years; men aged 45–60 years);^16^ and the German series comprised 902 patients who underwent surgery for glioma at the University of Bonn Medical Centre, between 1996 and 2008,^6^ with 2400 healthy individuals from the Heinz–Nixdorf Recall study serving as controls.^17^ The study was conducted with ethical review board approval. Written informed consent was obtained from all subjects. DNA was extracted from EDTA-venous bloods using conventional methodologies and quantified using PicoGreen (Invitrogen Corp., Carlsbad, CA, USA).

### The exome array

Briefly, the Illumina HumanExome-12v1_A Beadchip (Illumina, San Diego, CA, USA) includes 247 870 markers focused on protein-altering variants identified from whole-exome sequencing DNA from >12 000 individuals of multiple ethnicities and with multiple diseases/traits. In addition to 203 310 PAVs, the array also features 4761 GWAS trait-associated SNPs, 2061 HLA tags, 3015 ancestry-informative markers, 4896 identity-by-descent estimation markers and 4139 random synonymous SNPs. Comprehensive details about the exome array are available at http://genome.sph.umich.edu/wiki/Exome_Chip_Design.

### Exome array data availability

Illumina HumanExome-12v1_A Beadchip array genotypes for individuals from the 1958BC are available from the European Genome-phenome Archive (EGA, http://www.ebi.ac.uk/ega/) under accession number EGAD00010000234. Similarly, array genotypes and phenotypes for the remaining datasets in this study have been deposited to EGA and are available under accession number EGAS00001001258.

### Genotyping and quality control

Genotyping was conducted using Illumina HumanExome-12v1_A Beadchips in accordance with the manufacturer's recommendations (Illumina). Calling of genotypes was performed using Illumina GenomeStudio version 2011.1 software. Cluster boundaries were determined by calling study samples simultaneously. Probes were excluded if monomorphic in all datasets, had a call rate <0.99 in cases/controls in a series, the difference in uncalled genotypes between cases and controls was statistically significant (*P*<0.05), if Hardy–Weinberg in controls *P*<0.001, or if non-autosomal (Supplementary Table 1). Samples were excluded if the call rate was <0.99, outlying heterozygosity (>3 SD), or if a discrepancy was observed between manifest sex and X-chromosome genotype. To assess the fidelity of genotyping we examined the concordance in 493 individuals from the 1958BC,^15^ which had also been sequenced^18^ using TruSeq capture in conjunction with Illumina HiSeq2000 technology, and a GATK2 ^ref. 19^ pipeline according to best practices.^20, 21^ Genotypes were compared at genomic positions for which allele codings could be unambiguously assigned, excluding 257A/T and C/G SNPs with MAF>0.40.

### Statistical and bioinformatic analysis

The main statistical and bioinformatics analyses were performed using PLINK v1.07^(ref. 22)^ (Cambridge, MA, USA) and R v3.0 software (Vienna, Austria). Using the EIGENSOFT v4.2 smartpca package^23, 24^ (Cambridge, MA, USA) we performed PCA to ensure comparability of case and controls. Individuals with non-Western European ancestry were identified and excluded by merging case and control data with 1000 Genomes project data. 100 000 ld-pruned post-QC probes were used to compute eigenvectors in each cohort. Samples exhibiting significant deviations (6 SD) from the main case/control cluster up to the first 10 eigenvectors were classified as outliers and flagged for exclusion. Outlying population structure on the pruned data set was examined using fastSTRUCTURE^25^ if subsequent non-comparability was apparent between cases and controls. For first-degree relative pairs, the control from a case–control pair was removed; otherwise, the individual with the lower call rate was excluded. Associations were tested under an additive model. The adequacy of the case–control matching in each series and the possibility of differential genotyping of cases and controls was evaluated using quantile–quantile (Q–Q) plots of test statistics, restricting to variants with MAF>0.005 to derive reasonable inflation estimates. Meta-analysis *P*-values and odds ratios (ORs) were calculated from per-study logistic regression beta values, under a fixed-effects model. We used Cochran's *Q* statistic to test for heterogeneity; restricting the reporting of novel associations to those with *P*_het_ >0.05. We visually inspected genotype cluster plots for all reported variants. To explore variability in associations according to tumour histology, we derived ORs for all glioma, GBM and non-GBM. For the gene-based analysis, in addition to using the burden test which counts the number of minor alleles per gene per individual summed for all cases and controls, the sequence kernel association test (SKAT) was applied.^26^ Burden and SKAT gene-based tests were based on all post-QC non-monomorphic probes mapping to RefSeq genes imposing default weights and MAF<0.05. Tests were implemented in plink-seq v0.09, and adjusted for study-specific effects by incorporating study as a covariate (using covar option). A single-variant association was declared significant if *P*<1.40 × 10^−7^ (Bonferroni correction for 118,815 PAVs, three tumour types). Gene-based association tests were considered significant if *P*<2.49 × 10^−6^ (10 045 genes, two tumour types). The power of our study to demonstrate an association for alleles with different MAFs was calculated assuming a multiplicative model. In all analyses a *P*-value of 0.05 was considered as representing statistical significance, after adjustment for multiple testing. Gene-set enrichment analysis (GSEA) of pre-ranked SKAT *P*-values, was performed on gene sets catalogued by the MSigDB v4.0 database (updated 31 May 2013) using GSEA software^27^ adopting default settings. Linkage disequilibrium (LD) *r*^2^ metrics were estimated from UK10K whole-genome data. To restrict our analysis to genes with a higher likelihood of having a role in glioma on the basis of somatic mutation profile in tumours, we used MutSigCV version 1.4^ref. 28^ to identify genes harbouring more non-synonymous mutations than expected by chance given gene size, sequence context and mutation rate. Thresholding at false discovery rate *Q*<0.1 as advocated,^28^ MutSig scores were obtained for GBM and non-GBM tumours by interrogation of TCGA (The Cancer Genome Atlas) provisional data sets using cBioPortal.^29^ The Variant Effect Predictor (VEP; version 74)^30^ was used to predict impact of variants on canonical Ensembl gene transcripts and functional consequences of missense variants according to SIFT,^31^ PolyPhen-2^ref. 32^ and CONDEL.^33^ Computational modelling of the effect of amino acid changes on protein structure was carried out using the project HOPE server.^34^ To assess sequence conservation we used GERP^35^ and Phast_cons^36^ metrics.

### Quality control and array characteristics and performance

We submitted 2413 cases and 3099 controls for genotyping. Twelve cases and eight controls failed genotyping (call rate<0.95). Five hundred and nineteen cases and 807 controls were excluded for the following reasons: outlying heterozygosity in rare (47 cases, 28 controls) and common (44 cases, 10 controls) SNPs; duplicates/close relatives (15 cases, 16 controls); sex discrepancies (29 cases, 10 controls); and non-European ancestry (49 cases, 5 controls; Supplementary Table 1 and Supplementary Figure 1A). Genotypes from 5964 individuals were available from the 1958BC (UK) series. We further excluded 169 individuals because of personal history of cancer (105), outlying heterozygosity (16), sex discrepancy (22), duplicates/relatives (2) and non-European ancestry (24) (Supplementary Table 1). After excluding technical failures and imposing marker-level quality control, 90.2% of attempted markers were successfully genotyped (223 564/247 870). Concordance between genotype calls was assessed at 55 955 sites in the 493 individuals for whom exome-chip and whole-exome sequence data were available (Supplementary Table 2). Overall the concordance was: 99.7%, with 96.5%, 96.0% and 99.8% when comparing minor homozygotes, heterozygotes and major homozygotes respectively. Restricting our analysis to 219 771 autosomal probes, 84 502 markers were monomorphic (38.5%). Q–Q plots of association test statistics showed there was minimal inflation in the UK and French series (*λ*=1.04 and 1.05; Supplementary Figure 2A and B). In the German series, *λ* was 1.17 (Supplementary Figure 2C). Using fastSTRUCTURE^25^ to impose two populations within the German series and retaining only individuals with >80% membership of the larger population (2083 individuals, 488 cases and 797 controls; Supplementary Table 1 and Supplementary Figure 1B) *λ* was 1.058 ensuring subsequent analysis was less biased by any ancestral discordance between cases and controls (Supplementary Figure 2D). Post-QC data on 1882 cases and 8079 controls were available for analysis.

## Results

### Single-variant associations

In total 135 269 variants (MAF>0.0) were taken forward for association testing in 1882 cases and 8079 controls. Genotypes for previously identified glioma GWAS risk SNPs or their proxies (ie, *r*^2^>0.8) were available for 5p15.33, 7p11.2, 8q24.21, 9p21.3, 11q23.3 and 20q13.33 risk loci.^6, 7, 8^ OR and tumour subtype-specific associations were consistent with those previously documented (Supplementary Table 3).

To assess the impact of recurrent variants exerting a putative effect on protein function, we restricted our analysis to 118 815 variants; 110 625 missense, 5324 splice-site altering, 2616 stop gain, 168 uRNA targets and 82 indels. The MAF distribution was highly skewed towards very low-frequency variants (Supplementary Figure 3), with 80.4% (*n*=95 488) of variants successfully genotyped having a control MAF≤0.005; 4.0% (*n*=4,764) with MAF=0.05–0.01; 6.4% (*n*=7546) with MAF=0.01–0.05; and 9.3% (*n*=11 017) with MAF>0.05.

In the combined analysis of all PAVs the strongest association for risk of glioma was provided by rs593818 responsible for the XM_006722850.1(*CYP4F12*):c.1117A>G, p.(Ser373Gly) amino acid change (*P*=1.24 × 10^−5^), albeit non-significant on a genome-wide basis (Supplementary Table 4). Similarly in the stratified analysis no single variant showed a globally significant association with either GBM or non-GBM tumours (Supplementary Table 4).

**Figure 1:**
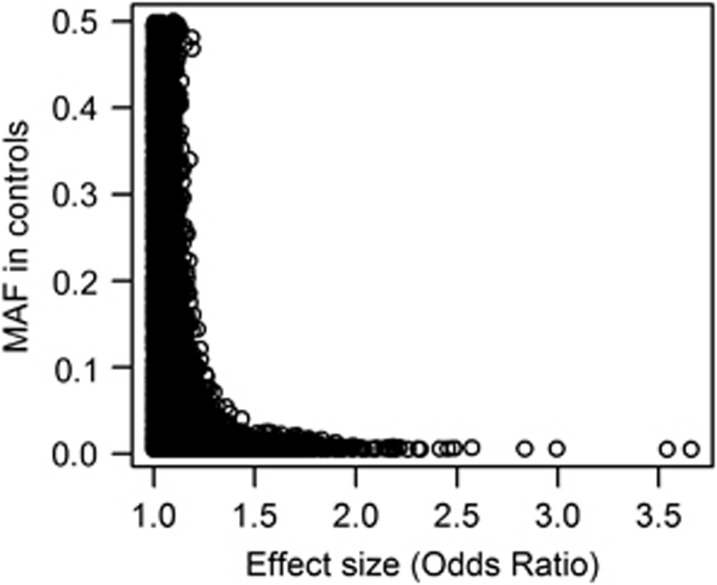
Relationship between effect size and minor allele frequency of PAVs.

Figure 1 shows the relationship between effect size (measured by OR, taking the reciprocal or ORs <1.0) and MAF for 118,815 PAVs, those SNPs characterized by low MAF tending to have a higher probability of conferring more substantive risks.

To restrict our analytical space, we analysed the data set incorporating information on the predicted functional consequences of these PAVs. Of the 104 321 PAVs genotyped by the exome array for which CONDEL annotations could be obtained, the majority (64.1%) are predicted to be neutral (*n*=66 841), and 35.9% deleterious (*n*=37 480). Fifteen PAVs predicted to be deleterious showed an association with glioma risk at the *P*<10^–3^ threshold (Table 1). To investigate whether PAVs predicted to be functionally deleterious were enriched for stronger effects on glioma risk, we compared the distribution of effect size (as measured by ORs) in the two CONDEL prediction categories (Table 2). There was strong evidence of a relationship between increasing effect size and prediction of the PAV being deleterious. For PAVs with control MAF>0.005 predicted to be deleterious there was an OR increase of 1.22 compared with neutral PAVs (95% confidence interval (CI): 1.19–1.26, *P*_trend_=2.29 × 10^–49^, Table 2). Overall, PAVs classified as damaging by CONDEL were 1.43-fold more likely to be associated with effect sizes ≥1.5 than PAVs classified as neutral (*P*=4.59 × 10^–4^, OR=1.43, 95% CI=1.17–1.74).

We further stratified our analysis to variants in genes that are significantly mutated in GBM and non-GBM glioma, as well as being nominally associated with glioma risk (*P*<0.05). This identified 11 variants also significantly associated with GBM and five with non-GBM glioma (Table 3). Of interest is NM_002168.3(*IDH2*):c.782G>A, p.(Arg261His) (rs118101777, non-GBM OR=3.21, *P*=7.7 × 10^−3^), which is predicted to be deleterious by CONDEL and is highly evolutionarily conserved (PhastCons=1.00, GERP=5.84).

A number of rare variants recognised to have pleiotropic effects on cancer risk are featured on the Illumina Exome Array (Table 4). For example, NM_000059.3(*BRCA2)*:c.9976A>T, p.(Lys3326Ter) (rs11571833), which increases breast and lung cancer risk,^10, 37^ NM_007194.3(*CHEK2*):c.470T>C, p.(Ile157Thr) (rs17879961), which increases breast cancer and CRC risk but decreases lung cancer risk,^10, 12, 38^ and NM_032043.2(*BRIP1*):c.139C>A, p.(Pro47Ala) (rs28903098), which has been implicated in familial breast and ovarian cancer.^39^ Given that such variants are *a priori* strong candidates for influencing the development of cancer, we examined the relationship between rs11571833, rs17879961 and rs28903098 and glioma (Table 5). For all glioma, *BRCA2* p.(Lys3326Ter) carrier status conferred an OR of 1.76 (*P*=0.0026), principally associated with GBM (OR=2.3, *P*=4.0x10^−4^). Although no association was shown for *CHEK2* p.(Ile157Thr), *BRIP1* p.(Pro47Ala) carrier status conferred an OR of 3.83 (*P*=0.048) (Table 4).

### Gene and gene-set-based tests

As the majority of individual variants typed are very rare (median MAF=3.7 × 10^−4^), we assessed the burden of 70 526 variants across 10 045 genes. *HARS2* showed an exome-wide significant association with GBM (Burden *P*=2.00 × 10^−6^, SKAT *P*=1.03 × 10^−5^, Table 5). Although not attaining exome-wide statistical significance, further gene-based tests revealed a number of genes that were both significantly mutated in glioma tumours as well as possessing a germline variant burden (Table 5).

To gain further insight into the nature of the biological pathways impacting on glioma susceptibility, we performed GSEA using SKAT association *P-*values (Supplementary Table 5). This revealed a number of gene sets that were positively or negatively enriched for genes associated with glioma (ie, *P*_*GSEA*_<0.05). GBM glioma showed positive enrichment for genes involved in amino acid and nucleotide metabolism, and non-GBM glioma showed positive enrichment for genes involved in cell growth and development, however the majority of gene sets had an FDR *Q*>0.25.

## Discussion

GWAS have become a powerful tool to identify susceptibility variants for cancer. However since the tagSNPs used in GWAS are generally not themselves candidates for causality, identification of the functional variant at a locus generally poses a significant challenge. An alternative approach is to target sequence variation, which *a priori*, is more likely to impact on disease status. Alleles that are functionally deleterious will tend to be selected against and thus underrepresented at high frequencies, an assertion supported by the observation of a relationship between putative functionality and MAF. Hence, it can be argued that at least some of the variants impacting on cancer risk including glioma will be rare. Although the association between the rare variant *BRCA2*:c.9976A>T, p.(Lys3326Ter) and glioma did not attain statistical significance such an assertion is supported by the established relationship between *CHEK2*:c.1100delC, p.(Thr367Metfs) and *MUTYH:*c.536A>G, p.(Tyr179Cys) and *MUTYH*:c.1187G>A, p.(Gly396Asp) variants which influence the risk of breast and CRC respectively.^12, 13^

To our knowledge we have conducted the largest study of the relationship between recurrent PAVs and glioma risk to date. Population stratification is a source of bias in association studies, and although adjustment of test statistics for principal components generated on common SNPs can be applied to genome scans, confounding of rare variants in spatially structured populations is not necessarily corrected by such methods.^40^ Hence a major strength of our study is that it is based on three independent case–control series, thereby minimising biases as a consequence of spatial differences within one data set impacting on conclusions.

No single-variant associations with glioma attained statistical significance after correction for multiple testing. However, we did observe a significant association between variant effect size and predicted functional effect. In this study we have been limited to detecting alleles conferring ORs of 1.6 provided MAF >0.05 (80% power stipulating *P*<10^–7^) or those with frequencies of ~0.01 conferring ORs >2.5. Hence it is possible that PAVs do have an appreciable contribution to glioma risk but at lower individual effect sizes than previously anticipated, therefore requiring much larger case–control sample sets than we have used herein to identify them.

Testing for a burden of PAVs across genes revealed a significant association between *HARS2* and GBM. *HARS2* encodes a mitochondrial histidyl tRNA synthetase, mutation of which causes ovarian dysgenesis and sensorineural hearing loss.^41^ Although not attaining an exome-wide significant burden of germline variants, additionally of note is *CDH18* and GBM risk. *CDH18* is also significantly mutated in GBM tumours and encodes a cadherin protein involved in cell–cell adhesion. The gene is expressed specifically in the nervous system and has been proposed to regulate neural morphogenesis.^42^

By restricting our analysis to genes implicated in glioma by virtue of somatic mutation or variants recognised to increase risk of other cancers, we constrained the multiple testing problem and upweighted the prior probability for association with glioma. From these analyses we have provided evidence to implicate *BRCA2* p.(Lys3326Ter) as well as *IDH2* p.(Arg261His) as determinants of glioma risk. *IDH2* encodes for the mitochondrial NAD(+)-dependent isocitrate dehydrogenase which is involved in the citric acid cycle.^43^ While *IDH2* p.(Arg261His) is not mutated in glioma, *IDH1* or *IDH2* are commonly mutated in glioma tumours and always involve the arginine residue.^44^ *IDH2* is in chromosome 15q26.1, the location of a previously reported glioma linkage peak.^45^ Modelling of the *IDH2* p.(Arg261His) change is shown in Supplementary Figure 4. This amino acid change is predicted to disrupt several salt bridge interactions, which may affect protein activity.

In our study, none of the PAVs genotyped in any of the previously identified glioma GWAS regions showed evidence of association with glioma (*n*=240; *P*>1.37 × 10^−3^). While accepting that we are constrained by the content of PAVs on the array, this argues against a rare coding variant that is tagged by a SNP contributing significantly to any of the GWAS signals identified.

While aiming to provide a comprehensive survey of recurrent PAVs it is apparent from our analysis that there are a number of issues that will impact on the utility of the Illumina Exome Array. Firstly, a high proportion of the featured SNPs are either monomorphic in Europeans or have a MAF <0.005. Secondly, as illustrated by comparison with data from the UK10K sequencing project, 22% of missense variants with allele counts>5 are not featured on the array (11 894 of 54 463 variants; Supplementary Table 6). Additionally, only ~36% of PAVs on the array are predicted to be functionally deleterious. Finally, indels are not well represented on the array. Collectively, these observations cast doubt on the ability of the array to provide a comprehensive assessment of the contribution of PAVs to disease risk, highlighting the value of sequence-based approaches to discover new disease variants.

In conclusion, there is increasing evidence that cancer susceptibility is in part mediated through low-frequency variants affecting the amino acid sequence of expressed proteins. Hence genome scans of PAVs represent a complementary strategy to identify disease-causing variants compared to scans based on tagSNPs. Strategies to lessen the multiple testing burden by restricting analysis to PAVs with higher priors affords an opportunity to maximise study power.

## Websites

R Core Team (2013) R: A language and environment for statistical computing. R Foundation for Statistical Computing, Vienna, Austria: URL http://www.R-project.org/; Illumina: http://www.illumina.com; Exome chip design: http://genome.sph.umich.edu/wiki/Exome_Chip_Design; Plink: http://pngu.mgh.harvard.edu/~purcell/plink/; Plink seq: https://atgu.mgh.harvard.edu/plinkseq/; cBioPortal for Cancer Genomics: http://www.cbioportal.org; The Cancer Genome Atlas project: http://cancergenome.nih.gov; UK10K: http://www.uk10k.org/; Wellcome Trust Case Control Consortium (WTCC2): http://www.wtccc.org.uk/; GERP: http://snp.gs.washington.edu/SeattleSeqAnnotation134/; Phast_cons: http://genome.ucsc.edu/cgi-bin/hgGateway); Project HOPE server: http://www.cmbi.ru.nl/hope

## Figures and Tables

**Table 1:** PAVs classified as deleterious by CONDEL associated with glioma risk at *P* <10^−3^

					*All glioma N*_c__ase/control_=*1882/8079*		*GBM N*_c__ase_=*771*		*Non-GBM N*_case_=*928*	
	*HGVS genomic*		*Allele frequency*							
*dbSNP rsid*	*Description*	*Gene*	*Case*	*Control*	P	*Odds ratio*	P	*Odds ratio*	P	*Odds ratio*
rs185338080	chr17:g.7329692C>T	*C17orf74*	0.00267	5.60 × 10^−4^	4.73 × 10^−5^	6.87 (2.71–18.4)	7.35x10^−6^	12.3 (4.11–37.0)	0.0374	4.29 (1.09–16.9)
rs200918780	chr2:g.103340351G>C	*MFSD9*	0.00213	7.43 × 10^−4^	9.78 × 10^−5^	8.80 (2.95–26.3)	0.00153	8.99 (2.31–34.9)	0.00109	9.62 (2.47–37.4)
rs144793260	chr5:g.52193318A>G	*ITGA1*	0.00133	3.09 × 10^−4^	1.08 × 10^−4^	25.6 (4.96–134)	0.00241	20.9 (2.93–149)	0.00193	22.4 (3.14–159)
rs200058353	chr15:g.42983771C>G	*KIAA1300*	0.00133	1.86 × 10^−4^	1.08 × 10^−4^	25.6 (4.96–132)	0.0560	10.4 (0.94–115)	1.157 × 10^−5^	45.1 (8.22–247)
rs41281932	chr9:g.100116970A>G	*KIAA1529*	0.00478	0.00173	1.22 × 10^−4^	3.48 (1.84–6.57)	2.68 × 10^−4^	4.15 (1.93–8.92)	0.0316	2.77 (1.09–7.02)
rs2229388	chr8:g.16012648G>C	*MSR1*	0.0689	0.0545	1.25 × 10^−4^	1.36 (1.16–1.59)	0.00144	1.41 (1.14–1.74)	0.0304	1.26 (1.02–1.56)
rs140963213	chr11:g.67814983G>A	*TCIRG1*	0.00956	0.00464	1.41 × 10^−4^	2.33 (1.51–3.60)	0.0199	2.08 (1.12–3.87)	5.52 × 10^−4^	2.62 (1.52–4.52)
rs147288996	chr5:g.140057509C>T	*HARS*	0.00452	0.00167	2.08 × 10^−4^	3.28 (1.75–6.15)	2.83 × 10^−4^	3.93 (1.88–8.22)	0.00105	5.45 (1.98–15.0)
rs78805068	chr5:g.140308281C>T	*PCDHAC1*	0.00452	0.00161	2.14 × 10^−4^	3.38 (1.77–6.44)	1.91 × 10^−4^	4.11 (1.96–8.65)	0.00105	5.45 (1.98–15.0)
rs149397155	chr3:g.15298590C>T	*SH3BP5*	0.00213	0.00124	5.47 × 10^−4^	4.33 (1.89–9.93)	7.16 × 10^−4^	5.55 (2.06–15.0)	0.253	2.35 (0.54–10.1)
rs118101777	chr15:g.90630704C>T	*IDH2*	0.00425	0.00155	5.50 × 10^−4^	3.11 (1.63–5.91)	0.00302	3.41 (1.52–7.67)	0.00767	3.21 (1.36–7.57)
rs201460298	chr7:g.111375137G>C	*DOCK4*	0.00159	1.86 × 10^−4^	6.60 × 10^−4^	12.4 (2.91–52.8)	1.63 × 10^−4^	31.5 (5.24–189)	0.0111	10.5 (1.71–64.8)
rs139894978	chr7:g.156755735C>G	*NOM1*	0.00266	0.00192	8.00 × 10^−4^	3.43 (1.67–7.06)	0.0551	2.80 (0.98–7.99)	8.38 × 10^−4^	4.52 (1.87–11.0)
rs74720216	chr17:g.8159165G>A	*PFAS*	0.0122	0.0182	8.84 × 10^−4^	0.57 (0.40–0.79)	0.0714	0.66 (0.42–1.04)	0.0165	0.58 (0.37–0.91)
rs2822432	chr21:g.15516948C>T	*LIPI*	0.363	0.331	1.00 × 10^−3^	1.15 (1.06–1.25)	0.0460	1.12 (1.00–1.26)	0.00145	1.20 (1.07–1.34)

*P*-values and odds ratios (ORs) estimated from fixed-effects meta-analysis of logistic regression beta values, assuming an additive model. Variants are ordered by glioma association *P*-value. HGVS, human genome variation society. ORs and allele frequencies derived with respect to underlined allele in HGVS genomic description. All genomic variant descriptions based on genome build hg19.

**Table 2:** Classification of PAVs with MAF>0.005 by Condel prediction, stratified by effect size in glioma

	*Condel prediction*			
*Effect size*^a^	*Neutral*	*Deleterious*^b^	*Unknown*	*Total*
<1.05	6687 (44.2%)	1762 (34.9%)	1389 (44.9%)	9838 (42.3%)
1.05–1.10	3603 (23.8%)	1125 (22.3%)	788 (25.5%)	5516 (23.7%)
1.10–1.20	2648 (17.5%)	1086 (21.5%)	500 (16.2%)	4234 (18.2%)
1.20–1.50	1868 (12.4%)	932 (18.5%)	352 (11.4%)	3152 (13.6%)
1.50–2.00	287 (1.9%)	136 (2.7%)	59 (1.9%)	482 (2.1%)
2.00–3.00	19 (0.1%)	8 (0.2%)	6 (0.2%)	33 (0.1%)
>3.00	0 (0.0%)	1 (0.02%)	1 (0.03%)	2 (0.0%)
Total	15 112	5050	3095	23 257

a Measured by odds ratio (taking the reciprocal for OR<1.0).

b *P*_trend_=2.29 × 10^−49^ (Deleterious vs neutral; OR_trend_=1.22, 95% CI: 1.19–1.26).

**Table 3:** Protein altering variants (PAVs) in genes significantly mutated in GBM and non-GBM Gliomas

					*GBM*		*Non-GBM*	
*dbSNP rsid*	*HGVS genomic description*	*Gene*	*Mutsig Q*	*Control allele frequency*	P	*Odds ratio*	P	*Odds ratio*
*GBM*								
rs72658163	chr7:g.94049588G>A	*COL1A2*	0.0157	0.00204	0.0191	3.00 (1.20–7.52)		
rs11569729	chr4:g.70592915G>A	*SULT1B1*	0.00279	0.00159	0.0208	3.22 (1.19–8.68)		
rs121908919	chr2:g.167138296T>C	*SCN9A*	0.0623	0.00233	0.0229	2.76 (1.15–6.64)		
rs12364102	chr11:g.56949691G>A	*LRRC55*	0.0223	0.126	0.0244	0.82 (0.69–0.97)		
rs201984007	chr2:g.167128917A>G	*SCN9A*	0.0623	5.10 × 10^−4^	0.0263	5.97 (1.23–28.9)		
rs112884419	chr1:g.158582637C>A	*SPTA1*	1.85 × 10^−9^	0.00210	0.0318	2.64 (1.09–6.38)		
rs144312303	chr5:g.67586574G>T	*PIK3R1*	0.000	2.84 × 10^−4^	0.0320	20.8 (1.30–334)		
rs149858889	chr7:g.94050334C>T	*COL1A2*	0.0157	1.70 × 10^−4^	0.0336	13.6 (1.23–150)		
rs140336416	chr7:g.93116243A>G	*CALCR*	0.0079	2.27 × 10^−4^	0.0336	13.6 (1.23–150)		
rs140857588	chr5:g.19571925T>C	*CDH18*	4.15 × 10^−5^	2.27 × 10^−4^	0.0401	5.93 (1.08–32.5)		
rs71428908	chr2:g.167160752G>C	*SCN9A*	0.0623	0.00181	0.0440	2.70 (1.03–7.10)		

*Non-GBM*								
rs12442879	chr15:g.57524982G>A	*TCF12*	7.04 × 10^−4^	0.0323			2.35 × 10^−4^	1.65 (1.26–2.14)
rs118101777	chr15:g.90630704C>T	*IDH2*	2.01 × 10^−12^	0.00147			0.00767	3.21 (1.36–7.57)
rs72470545	chr2:g.74759825G>A	*HTRA2*	1.01 × 10^−4^	0.00335			0.0126	2.32 (1.20–4.49)
rs140596855	chr15:g.90628584C>T	*IDH2*	2.01 × 10^−12^	2.84 × 10^−4^			0.0284	22.3 (1.39–357)
rs114905908	chr4:g.162577630A>T	*FSTL5*	0.0078	3.40 × 10^−4^			0.0493	11.1 (1.01–123)

Shown are genes with meta-analysis *P-*values<0.05 and MutSig false discovery rate Q values<0.1 for the relevant tumour type. HGVS, human genome variation society. Odds ratios and allele frequencies derived with respect to underlined allele in HGVS genomic description. All genomic variant descriptions based on genome build hg19.

**Table 4:** Protein altering variants (PAVs) previously implicated in multiple cancers

			*Allele Frequency*		*All Glioma*		*GBM*		*Non-GBM*		
*dbSNP rsid*	*Gene*	*HGVS genomic description*	*Case*	*Control*	P	*Odds ratio*	P	*Odds ratio*	P	*Odds ratio*	*Reference*
rs11571833	*BRCA2*	chr13:g.32972626A>T	0.012	0.0085	0.0026	1.76 (1.22–2.53)	4.0 × 10^−4^	2.30 (1.45–3.64)	0.060	1.67 (0.98–2.91)	(Michailidou *et al*;^37^ Wang *et al*, ^10^)
rs17879961	*CHEK2*	chr22:g.29121087 A>G	0.0041	0.0030	0.83	0.93 (0.49–1.79)	0.78	1.13 (0.50–2.53)	0.88	0.92 (0.33–2.61)	(Han *et al*,^38^ Wang *et al*,^10^)
rs28903098	*BRIP1*	chr17:g.59937223G>C	0.0011	5.8 × 10^−4^	0.048	3.83 (1.01–14.5)	0.037	5.22 (1.10–24.7)	0.34	2.78 (0.35–22.4)	(Cantor *et al.*,^39^)

HGVS, human genome variation society. Odds ratios and allele frequencies derived with respect to underlined allele in HGVS genomic description. All genomic variant descriptions based on genome build hg19.

**Table 5:** Genes possessing significant burden of germline variants as well as being significantly mutated in GBM and non-GBM tumours

		*Germline (exome-array)*				
*Gene*	*Variants*	*P*_*Burden*_	*Rank*_*Burden*_	*P*_*SKAT*_	*Rank*_*SKAT*_	*Tumour* *Mutsig Q*
*GBM*						
* HARS2*	7	2.00 × 10^−6^	**1**	1.03 × 10^−5^	**4**	>0.1
* CRAMP1L*	7	4.84 × 10^−5^	**3**	5.79 × 10^−5^	**9**	>0.1
* RBM47*	3	2.29 × 10^−4^	**8**	1.57 × 10^−5^	**6**	>0.1
* SLC26A6*	8	7.84 × 10^−5^	**6**	6.71 × 10^−5^	**10**	>0.1
* CDH18*	7	0.00672	119	0.116	1525	**4.15** × **10**^−**5**^
* ZPBP*	2	0.0414	615	0.154	2021	**0.0116**
* ABCB1*	14	0.0180	282	0.235	3098	**0.0154**
* SEMA3E*	3	0.0185	290	0.0772	1054	**0.0209**
* DYNC1I1*	5	9.57 × 10^−4^	24	0.00837	181	**0.071**

*Non-GBM*						
* CPM*	5	1.05 × 10^−4^	**18**	3.58 × 10^−4^	**11**	>0.1
* DYNC1I1*	5	0.028	517	0.0201	289	**2.01** × **10**^−**12**^
* HTRA2*	2	0.0103	213	0.0300	412	**1.01** × **10**^−**4**^
* TCF12*	7	4.71 × 10^−4^	**17**	0.000142	**8**	**7.04** × **10**^−**4**^
* PTPN11*	2	0.275	2816	0.0442	600	**0.0383**

Shown are genes with *P*<0.05 in at least one germline burden test and which either (1) are ranked in the top 20 most associated genes in both tests (highlighted in bold) or (2) have a Mutsig Q score <0.1 for the relevant tumour subtype (indicating significantly mutated genes, highlighted in bold).
