# Supplementary material for: Search for new loci and low-frequency variants influencing glioma risk by exome-array analysis
Source: Eur J Hum Genet. 2015 Aug 12;24(5):717–24. doi: 10.1038/ejhg.2015.170 (PMC4677454; doi:10.1038/ejhg.2015.170)
Supplement: Supplementary Figure 1 [file ejhg2015170x1.docx]

**A**


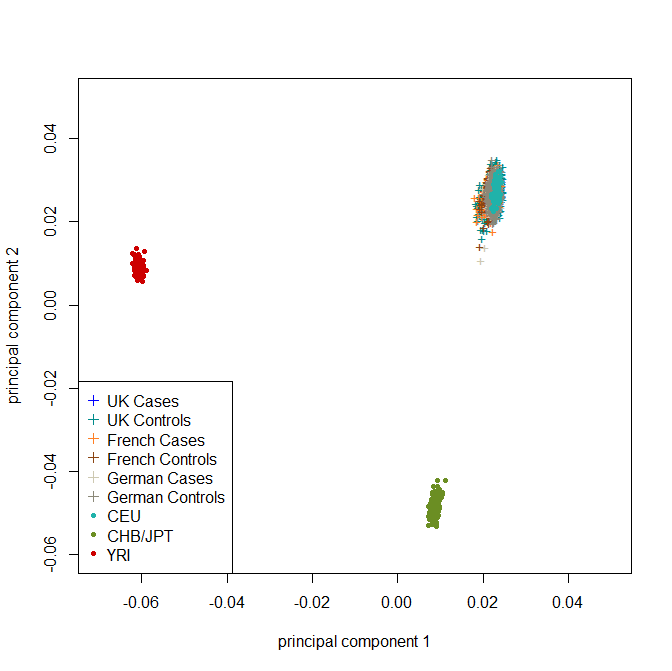


**B**


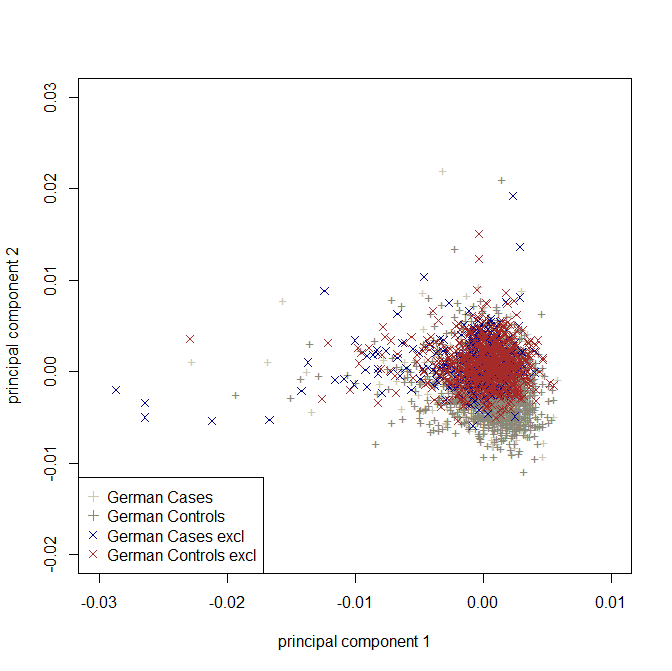


**Supplementary Figure 1:** **PCA plots of the population ancestry of cases and controls**. (a) Identification of individuals of non-European ancestry in the three series (HapMap CEU individuals are plotted in blue, CHB+JPT are plotted in green, YRI individuals are plotted in red); (b) PCA plot of German cases and controls. Red and blue crosses indicate german controls and cases excluded by fastSTRUCTURE respectively.
