# Supplementary material for: Search for new loci and low-frequency variants influencing glioma risk by exome-array analysis
Source: Eur J Hum Genet. 2015 Aug 12;24(5):717–24. doi: 10.1038/ejhg.2015.170 (PMC4677454; doi:10.1038/ejhg.2015.170)
Supplement: Supplementary Figure 2 [file ejhg2015170x2.docx]

**^A^**

^
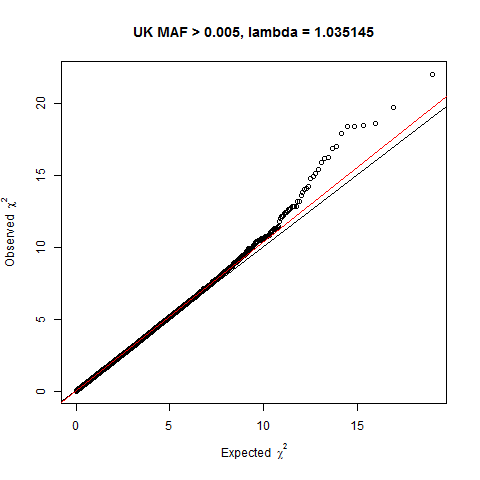
^

**^B^**

^
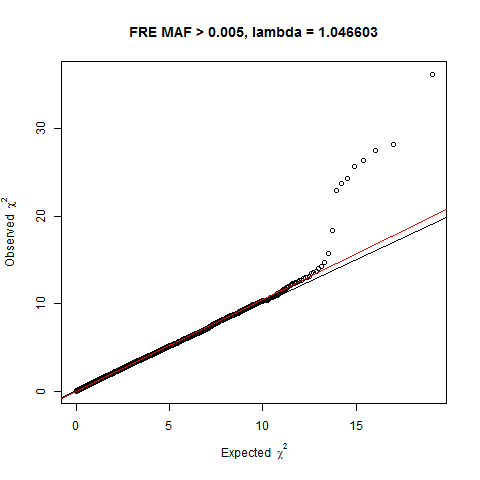
^

**^C^**

^
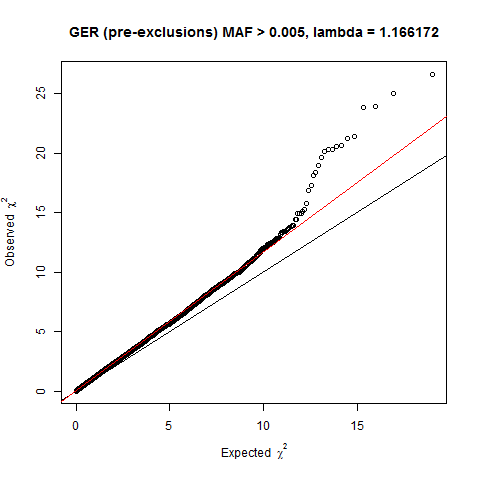
^

**D**

^
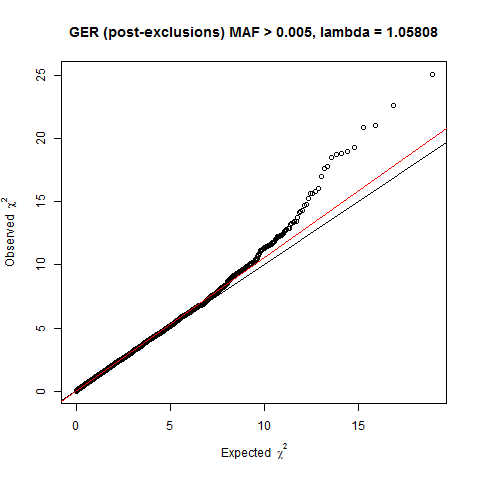
^

**Supplementary Figure 2: Q-Q plots of observed association χ^2^ values (y-axis) against expected from χ^2^ values (x-axis)**. Inflation λvalues calculated from the median chi-squared statistic. For each case-control cohort, only probes with MAF > 0.005 included. Black line is y=x, red line is linear fit. (a) UK series; (b) French series; (c) German series before exclusions; (d) German series after exclusions.
