# Supplementary material for: Search for new loci and low-frequency variants influencing glioma risk by exome-array analysis
Source: Eur J Hum Genet. 2015 Aug 12;24(5):717–24. doi: 10.1038/ejhg.2015.170 (PMC4677454; doi:10.1038/ejhg.2015.170)
Supplement: Supplementary Figure 4 [file ejhg2015170x4.docx]

**A**


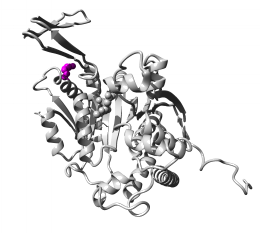


**B**


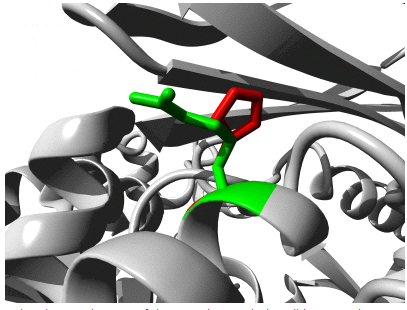


**Supplementary Figure 4. Structural modelling of the *IDH2* p.Arg261His amino acid change.** Modelling of the amino acid change was carried out using the HOPE server^34^. (a) *IDH2* in protein ribbon-presentation. *IDH2* p.Arg261His is indicated in magenta. (b) Close-up of *IDH2* p.Arg261His. *IDH2* is coloured grey, the side chains of the wild-type Arginine and mutant Histidine residue are shown and coloured green and red respectively.
