# Supplementary material for: Search for new loci and low-frequency variants influencing glioma risk by exome-array analysis
Source: Eur J Hum Genet. 2015 Aug 12;24(5):717–24. doi: 10.1038/ejhg.2015.170 (PMC4677454; doi:10.1038/ejhg.2015.170)
Supplement: Supplementary Table 1 [file ejhg2015170x5.docx]

| **a**. **Sample QC** |  | | |  | | |  |  | |  |  |  |  | |  |  |
| --- | --- | --- | --- | --- | --- | --- | --- | --- | --- | --- | --- | --- | --- | --- | --- | --- |
|  | **UK series** | | | | | **French series** | | | | **German series** | | **Total** | | | |  |
|  | **Cases** | | **Controls** | | | **Cases** | | **Controls** | | **Cases** | **Controls** | **Cases** | | **Controls** | |  |
| **pre-QC** | 605 | | 5,964 | | | 906 | | 699 | | 902 | 2,400 | 2,413 | | 9,063 | |  |
| **Failed genotyping** | 9 | | n/a | | | 0 | | 0 | | 3 | 9 | 12 | | 8 | |  |
| **Heterozygosity outlier (MAF > 0.01)** | 11 | | 14 | | | 16 | | 2 | | 17 | 8 | 44 | | 24 | |  |
| **Heterozygosity outlier (MAF < 0.01)** | 13 | | 2 | | | 12 | | 4 | | 22 | 24 | 47 | | 30 | |  |
| **Sex discrepancy** | 1 | | 22 | | | 15 | | 0 | | 13 | 10 | 195 | | 32 | |  |
| **Duplicates/relatives** | 0 | | 2 | | | 7 | | 0 | | 8 | 16 | 15 | | 18 | |  |
| **Non-European ancestry** | 1 | | 24 | | | 32 | | 4 | | 16 | 1 | 49 | | 29 | |  |
| **Outlying population substructure** | n/a | | n/a | | | n/a | | n/a | | 335 | 738 | 355 | | 738 | |  |
| **1958BC cancer** | n/a | | 105 | | | n/a | | n/a | | n/a | n/a | n/a | | 105 | |  |
| **TOTAL exclusions** | 35 | | 169 | | | 82 | | 10 | | 414 | 785 | 531 | | 964 | |  |
| **post-QC** | 570 | | 5,795 | | | 824 | | 689 | | 488 | 1,595 | 1,882 | | 8,079 | |  |
|  |  | | |  | | |  |  | |  |  |  |  | |  | |
| **b.** **Probe QC** | | | |  | | |  |  | |  |  |  |  | |  | |
|  | | **Removed** | | | **Remaining** | | | |  |  |  |  |  |  |  |  |
| **Pre-QC probes total** | | 247,480 | | | 247,480 | | | |  |  |  |  |  |  |  |  |
| **1. EXCLUSIONS (poorly performing probes)*** | | | | | | | | |  |  |  |  |  |  |  |  |
| **Case/Control CR < 99%** | | 13,981 | | |  | | | |  |  |  |  |  |  |  |  |
| ***P_case/control missingness_* < 0.05** | | 22,123 | | |  | | | |  |  |  |  |  |  |  |  |
| **Control HWE *P* < 0.001** | | 1,921 | | |  | | | |  |  |  |  |  |  |  |  |
| **Total exclusions** | | 23,916 | | | 223,564 | | | |  |  |  |  |  |  |  |  |
| **2. FURTHER FILTERING (for association analysis)** | | | | | | | | |  |  |  |  |  |  |  |  |
| **Non-autosomal** | | 5,574 | | | 219,771 | | | |  |  |  |  |  |  |  |  |
| **Monomorphic** | | 84,502 | | | 135,269 | | | |  |  |  |  |  |  |  |  |
| **Non protein-altering** | | 16,454 | | | 118,815 | | | |  |  |  |  |  |  |  |  |
| **Post-QC probes total** | | 130,446 | | | 118,815 | | | |  |  |  |  |  |  |  |  |

**Supplementary Table 1. Details of quality control (QC) for Glioma exome array genotype data.**

(a) Sample exclusions applied in hierarchy from top to bottom (e.g. sample excluded at first instance not considered by additional criteria). (b) - Probe QC exclusions and filters. HWE, hardy-weinberg equilibrium; MAF, minor allele frequency.

*Probe exclusions not applied sequentially, therefore total number of exclusions is actually less than the sum of probes removed when considering criteria individually.
