# Supplementary material for: Search for new loci and low-frequency variants influencing glioma risk by exome-array analysis
Source: Eur J Hum Genet. 2015 Aug 12;24(5):717–24. doi: 10.1038/ejhg.2015.170 (PMC4677454; doi:10.1038/ejhg.2015.170)
Supplement: Supplementary Table 2 [file ejhg2015170x6.docx]

|  |  | **Exome Sequence Genotype calls** | | | |
| --- | --- | --- | --- | --- | --- |
|  |  | **AA** | **AB** | **BB** | **NC** |
| **Exome** | **AA** | 1,151,119 | 1,517 | 39 | 50,198 |
| **Array** | **AB** | 40,615 | 2,156,511 | 39,065 | 392,745 |
| **Genotype** | **BB** | 175 | 8,232 | 23,723,573 | 1,465 |
| **calls** | **NC** | 4,314 | 3,725 | 11,241 | 788 |

**Supplementary Table 2:** Concordance of genotype calls from 493 1958BC samples for which both Illumina Exome Array and whole-exome sequence data were available. AA, major homozygote; AB, heterozygote; BB, minor homozygote; NC, no-call.
