# Supplementary material for: Search for new loci and low-frequency variants influencing glioma risk by exome-array analysis
Source: Eur J Hum Genet. 2015 Aug 12;24(5):717–24. doi: 10.1038/ejhg.2015.170 (PMC4677454; doi:10.1038/ejhg.2015.170)
Supplement: Supplementary Table 3 [file ejhg2015170x7.docx]

|  |  |  |  |  |  | **All Glioma** | | **GBM** | | **non-GBM** | |
| --- | --- | --- | --- | --- | --- | --- | --- | --- | --- | --- | --- |
| **Lead SNP** | **Locus** | **Published**  **SNP** | **LD (r^2^)** | **HGVS genomic description** | **Control**  **AF** | ***P*** | **Odds ratio** | ***P*** | **Odds ratio** | ***P*** | **Odds ratio** |
| rs4295627 | 8q24 | rs4295627 | - | g.130685457T>G | 0.176 | 1.72x10^-11^ | 1.40 (1.27-1.54) | 0.167 | 1.10 (0.96-1.27) | 6.80x10^-19^ | 1.78 (1.57-2.02) |
| rs891835 | 8q24 | rs891835 | - | g.130491752T>G | 0.226 | 2.14x10^-8^ | 1.29 (1.18-1.41) | 0.066 | 1.13 (0.99-1.28) | 1.11x10^-9^ | 1.45 (1.29-1.63) |
|  |  |  |  |  |  |  |  |  |  |  |  |
| rs564398 | 9p21.3 | rs4977756 | 0.78 | g.22029547T>C | 0.425 | 3.21x10^-13^ | 1.34 (1.24-1.45) | 2.24x10^-11^ | 1.45 (1.30-1.62) | 4.98x10^-5^ | 1.25 (1.12-1.39) |
|  | 9p21.3 | rs4977756 | - | g.22068652G>A | 0.408 | 7.04x10^-13^ | 1.33 (1.23-1.44) | 9.89x10^-10^ | 1.40 (1.26-1.56) | 9.67x10^-6^ | 1.27 (1.14-1.42) |
|  |  |  |  |  |  |  |  |  |  |  |  |
| rs2853676 | 5p15.33 | rs2736100 | 0.22 | g.1288547T>C | 0.277 | 1.78x10^-8^ | 1.27 (1.17-1.39) | 2.93x10^-12^ | 1.50 (1.34-1.68) | 0.0453 | 1.12 (1.00-1.26) |
|  |  |  |  |  |  |  |  |  |  |  |  |
| rs2252586 | 7p11.2 | rs2252586 | - | g.54978924C>T | 0.287 | 1.64x10^-8^ | 1.27 (1.17-1.38) | 5.55x10^-8^ | 1.37 (1.22-1.54) | 8.09x10^-4^ | 1.21 (1.08-1.35) |
| rs11979158 | 7p11.2 | rs11979158 | - | g.55159349A>G | 0.173 | 5.32x10^-5^ | 0.79 (0.71-0.89) | 5.13x10^-6^ | 0.68 (0.58-0.80) | 0.333 | 0.93 (0.80-1.08) |
|  |  |  |  |  |  |  |  |  |  |  |  |
| rs11603023 | 11q23.3 | rs498872 | 0.04 | g.118486067T>C | 0.433 | 1.92x10^-8^ | 0.80 (0.73-0.86) | 0.309 | 0.95 (0.85-1.05) | 8.21x10^-13^ | 0.67 (0.60-0.75) |
| rs498872 | 11q23.3 | rs498872 | - | g.118477367A>G | 0.311 | 5.13x10^-4^ | 1.16 (1.07-1.25) | 0.271 | 1.07 (0.95-1.19) | 3.07x10^-6^ | 1.30 (1.16-1.44) |
|  |  |  |  |  |  |  |  |  |  |  |  |
| rs6010620 | 20q13.33 | rs6010620 | - | g.62309839A>G | 0.234 | 6.98x10^-6^ | 0.80 (0.72-0.88) | 2.50x10^-9^ | 0.64 (0.55-0.74) | 0.160 | 0.91 (0.80-1.04) |

**Supplementary Table 3: Evidence for association at previously reported glioma GWAS susceptibility loci.** Odds ratios (ORs) and *P*-values shown were obtained under a fixed-effects model. At each locus values are given for the previously reported SNP (or a proxy) and the lead SNP in this study. AF, allele frequency; HGVS, human genome variation society. ORs and allele frequencies derived with respect to underlined allele in HGVS genomic description. All genomic variant descriptions based on genome build hg19.
