# Supplementary material for: Search for new loci and low-frequency variants influencing glioma risk by exome-array analysis
Source: Eur J Hum Genet. 2015 Aug 12;24(5):717–24. doi: 10.1038/ejhg.2015.170 (PMC4677454; doi:10.1038/ejhg.2015.170)
Supplement: Supplementary Table 4 [file ejhg2015170x8.docx]

|  |  |  | **All glioma** | | | | **GBM** | | **non-GBM** | |
| --- | --- | --- | --- | --- | --- | --- | --- | --- | --- | --- |
|  | **HGVS genomic** |  | **Allele frequency** | |  |  |  |  |  |  |
| **dbSNP rsid** | **description** | **Gene** | **Case** | **Control** | ***P*** | **Odds Ratio** | ***P*** | **Odds Ratio** | ***P*** | **Odds Ratio** |
| **All glioma** |  |  |  |  |  |  |  |  |  |  |
| rs593818 | chr19:15807884A>G | *CYP4F12* | 0.51 | 0.47 | 1.24x10^-5^ | 1.19 (1.10-1.29) | 0.0572 | 1.11 (1.00-1.24) | 7.92x10^-6^ | 1.27 (1.14-1.41) |
| rs11040923 | chr11:6585007G>A | *DNHD1* | 0.51 | 0.48 | 2.32x10^-5^ | 1.19 (1.10-1.28) | 6.02x10^-4^ | 1.21 (1.09-1.35) | 0.00143 | 1.19 (1.07-1.32) |
| rs55708299 | chr10:105780285A>G | *SLK* | 0.0024 | 0.00087 | 4.36x10^-5^ | 6.33 (2.61-15.3) | 0.0143 | 4.83 (1.37-17.1) | 4.54x10^-5^ | 8.69 (3.07-24.6) |
| rs185338080 | chr17:7329692C>T | *C17orf74* | 0.0027 | 0.00056 | 4.73x10^-5^ | 6.87 (2.71-17.4) | 7.35x10^-6^ | 12.3 (4.11-37.0) | 0.0374 | 4.29 (1.09-16.9) |
| rs142824107 | chr11:48285686C>T | *OR4X1* | 0.0096 | 0.0054 | 4.90x10^-5^ | 2.32 (1.55-3.48) | 2.84x10^-7^ | 3.68 (2.24-6.05) | 0.0548 | 1.83 (0.99-3.39) |
| rs149351551 | chr9:77683924T>G | *C9orf95* | 0.0045 | 0.0019 | 7.40x10^-5^ | 3.83 (1.97-7.45) | 3.94x10^-6^ | 5.80 (2.75-12.2) | 0.109 | 2.32 (0.83-6.50) |
| rs200918780 | chr2:103340351G>C | *MFSD9* | 0.0021 | 0.00074 | 9.78x10^-5^ | 8.80 (2.95-26.3) | 0.00153 | 8.99 (2.31-34.9) | 0.00109 | 9.62 (2.47-37.4) |
|  |  |  |  |  |  |  |  |  |  |  |
| **GBM** |  |  |  |  |  |  |  |  |  |  |
| rs138650548 | chr7:72857133C>T | *BAZ1B* | 0.00133 | 0.000371 | 0.00400 | 6.19 (1.79-21.4) | 1.77x10^-5^ | 21.1 (5.24-84.6) | 0.866 | 1.27 (0.08-20.3) |
| rs192763050 | chr7:30961242A>G | *AQP1* | 0.0029 | 0.00223 | 0.00406 | 2.98 (1.42-6.29) | 1.88x10^-5^ | 5.58 (2.54-12.2) | 0.792 | 0.77 (0.10-5.63) |
| rs61745572 | chr19:897519C>T | *C19orf22* | 0.00425 | 0.00173 | 0.00196 | 2.96 (1.49-5.89) | 4.63x10^-5^ | 4.83 (2.26-10.3) | 0.843 | 1.16 (0.27-4.91) |
| rs199545411 | chr9:136905284C>G | *BRD3* | 0.00133 | 0.000433 | 4.13x10^-4^ | 8.54 (2.60-28.1) | 4.63x10^-5^ | 14.0 (3.94-50.0) | 0.226 | 3.71 (0.45-30.9) |
| rs79723119 | chr2:170013904A>C | *LRP2* | 0.0130 | 0.0122 | 0.132 | 1.31 (0.92-1.86) | 5.89x10^-5^ | 2.21 (1.50-3.26) | 0.678 | 0.87 (0.46-1.66) |
| rs144347065 | chr3:127395188G>A | *ABTB1* | 0.00717 | 0.00464 | 0.00361 | 2.05 (1.26-3.31) | 6.16x10^-5^ | 3.13 (1.79-5.47) | 0.750 | 1.14 (0.50-2.62) |
| rs10448108 | chr8:4809686C>T | *CSMD1* | 0.479 | 0.461 | 0.00173 | 1.13 (1.05-1.23) | 6.44x10^-5^ | 1.25 (1.12-1.39) | 0.426 | 1.04 (0.94-1.16) |
| rs199878911 | chr16:1709945C>T | *CRAMP1L* | 0.00824 | 0.00563 | 0.0171 | 1.71 (1.10-2.66) | 6.61x10^-5^ | 2.78 (1.68-4.60) | 0.954 | 0.98 (0.48-1.99) |
| rs143015514 | chr19:6926552C>T | *EMR1* | 0.00186 | 0.000495 | 0.006135 | 4.92 (1.57-15.4) | 7.05x10^-5^ | 10.1 (3.23-31.7) | 0.999 | 0.00 (0-Inf) |
| rs140988779 | chr19:56320924C>T | *NLRP11* | 0.00266 | 0.00179 | 0.0468 | 2.09 (1.01-4.33) | 7.63x10^-5^ | 4.59 (2.16-9.75) | 0.455 | 0.42 (0.04-4.07) |
| rs61743282 | chr2:128347710T>C | *MYO7B* | 0.00106 | 0.000496 | 0.00501 | 5.83 (1.70-20.0) | 7.96x10^-5^ | 12.0 (3.49-41.2) | 0.998 | 0.00 (0-Inf) |
| rs147489114 | chr17:72958366G>A | *C17orf28* | 0.00478 | 0.00161 | 8.90x10^-4^ | 3.81 (1.73-8.39) | 8.35x10^-5^ | 5.95 (2.45-14.5) | 0.0824 | 2.91 (0.87-9.69) |
| rs148378319 | chr9:111663930C>T | *IKBKAP* | 0.00691 | 0.00322 | 0.00670 | 2.02 (1.21-3.35) | 8.96x10^-5^ | 3.16 (1.78-5.63) | 0.362 | 1.44 (0.66-3.13) |
| rs186418869 | chr15:31233731T>C | *MTMR15* | 0.00186 | 0.000619 | 0.00302 | 5.80 (1.81-18.5) | 9.17x10^-5^ | 10.6 (3.25-34.6) | 0.196 | 4.89 (0.44-54.2) |
| rs148718670 | chr14:103574815G>A | *C14orf73* | 0.00664 | 0.00334 | 0.0641 | 1.69 (0.97-2.96) | 9.89x10^-5^ | 3.95 (1.98-7.90) | 0.929 | 0.96 (0.40-2.33) |
|  |  |  |  |  |  |  |  |  |  |  |
| **Non-GBM** |  |  |  |  |  |  |  |  |  |  |
| rs117802609 | chr20:44596262C>T | *ZNF335* | 0.0138 | 0.00755 | 4.27x10^-4^ | 1.96 (1.35-2.85) | 0.164 | 1.51 (0.84-2.70) | 6.08x10^-6^ | 2.80 (1.79-4.38) |
| rs1106639 | chr2:242690675G>A | *D2HGDH* | 0.301 | 0.263 | 0.00104 | 1.15 (1.06-1.26) | 0.706 | 1.02 (0.91-1.15) | 1.11x10^-5^ | 1.29 (1.15-1.44) |
| rs200058353 | chr15:42983771C>G | *KIAA1300* | 0.00133 | 0.000186 | 1.08x10^-4^ | 25.6 (4.96-132) | 0.0560 | 10.4 (0.94-115) | 1.16x10^-5^ | 45.1 (8.22-247) |
| rs117293522 | chr7:90896078A>G | *FZD1* | 0.00239 | 0.000743 | 0.00297 | 4.19 (1.63-10.8) | 0.313 | 2.12 (0.48-9.86) | 2.55x10^-5^ | 10.8 (3.57-32.8) |
| rs3789044 | chr1:204589101G>A | *LRRN2* | 0.252 | 0.227 | 0.00249 | 1.15 (1.05-1.26) | 0.901 | 0.99 (0.87-1.13) | 3.11x10^-5^ | 1.29 (1.14-1.45) |
| rs11588857 | chr1:204587047G>A | *LRRN2* | 0.242 | 0.218 | 0.00560 | 1.14 (1.04-1.25) | 0.663 | 0.97 (0.85-1.11) | 3.86x10^-5^ | 1.29 (1.14-1.45) |
| rs2235937 | chr1:29631909A>G | *PTPRU* | 0.252 | 0.223 | 0.000459 | 1.18 (1.07-1.29) | 0.166 | 1.09 (0.96-1.24) | 4.24x10^-5^ | 1.28 (1.14-1.45) |
| rs2229860 | chr7:103205827G>C | *RELN* | 0.00877 | 0.00495 | 0.00781 | 1.81 (1.17-2.79) | 0.816 | 1.10 (0.51-2.37) | 4.26x10^-5^ | 2.85 (1.73-4.71) |
| rs201532961 | chr17:72874476T>C | *FADS6* | 0.00186 | 0.000495 | 0.00496 | 5.84 (1.71-20.0) | 0.999 | 0.00 (0-Inf) | 5.01x10^-5^ | 12.9 (3.74-44.2) |
| rs149405381 | chr13:32371409G>A | *RXFP2* | 0.00372 | 0.00204 | 0.00569 | 2.61 (1.32-5.14) | 0.348 | 1.85 (0.51-6.66) | 6.24x10^-5^ | 4.53 (2.16-9.48) |
| rs202000786 | chr1:1904413T>C | *KIAA1751* | 0.00239 | 0.00198 | 0.00962 | 2.83 (1.29-6.22) | 0.742 | 0.72 (0.10-5.27) | 6.52x10^-5^ | 5.48 (2.38-12.6) |
| rs150175049 | chr17:62290191C>T | *TEX2* | 0.00372 | 0.00309 | 0.0233 | 2.07 (1.10-3.86) | 0.958 | 1.04 (0.25-4.32) | 6.73x10^-5^ | 4.14 (2.06-8.32) |
| rs117738342 | chr16:66880957A>T | *CA7* | 0.00611 | 0.00371 | 0.00385 | 2.18 (1.29-3.70) | 0.708 | 1.22 (0.43-3.43) | 8.05x10^-5^ | 3.56 (1.89-6.68) |

**Supplementary Table 4: Protein altering variants (PAVs) associated with Glioma risk.** *P* values and odds ratios obtained under a fixed-effect meta-analysis of logistic regression beta values from the three case-control series, assuming an additive model. Shown are associations with *P* < 10^-4^ for risk of all glioma, GBM and non-GBM tumours. HGVS, human genome variation society. ORs and allele frequencies derived with respect to underlined allele in HGVS genomic description. All genomic variant descriptions based on genome build hg19.
