# Supplementary material for: Search for new loci and low-frequency variants influencing glioma risk by exome-array analysis
Source: Eur J Hum Genet. 2015 Aug 12;24(5):717–24. doi: 10.1038/ejhg.2015.170 (PMC4677454; doi:10.1038/ejhg.2015.170)
Supplement: Supplementary Table 5 [file ejhg2015170x9.docx]

| **Gene set** | **Function** | ***N* Genes** | **ES** | **NES** | ***P_GSEA_*** | **FDR *q*** | **FWER *q*** |
| --- | --- | --- | --- | --- | --- | --- | --- |
| **All glioma positive enrichment** |  |  |  |  |  |  |  |
| SABATES_COLORECTAL_ADENOMA_UP | Cancer-related genes | 76 | 0.22 | 2.28 | 0.00 | 1.00 | 0.97 |
| GSE3982_EOSINOPHIL_VS_CENT_MEMORY_CD4_TCELL_DN | Immune regulation | 103 | 0.18 | 2.20 | 0.00 | 1.00 | 0.99 |
| V$AP2GAMMA_01 | Development/retinoic acid regulation | 131 | 0.16 | 2.18 | 0.00 | 1.00 | 1.00 |
| WIERENGA_STAT5A_TARGETS_UP | Cell-fate decision making | 100 | 0.18 | 2.18 | 0.00 | 1.00 | 1.00 |
| KEGG_PROXIMAL_TUBULE_BICARBONATE_RECLAMATION | Blood acidity level | 15 | 0.46 | 2.09 | 0.00 | 1.00 | 1.00 |
| GSE13306_LAMINA_PROPRIA_VS_SPLEEN_TREG_DN | Immune regulation | 111 | 0.17 | 2.06 | 0.00 | 1.00 | 1.00 |
| GSE13484_12H_VS_3H_YF17D_VACCINE_STIM_PBMC_DN | Immune regulation | 90 | 0.19 | 2.05 | 0.00 | 1.00 | 1.00 |
| MODULE_206 | Cancer-related genes | 79 | 0.20 | 2.04 | 0.00 | 1.00 | 1.00 |
| PID_IL27PATHWAY | Immune regulation | 16 | 0.42 | 2.03 | 0.00 | 1.00 | 1.00 |
| LEIN_OLIGODENDROCYTE_MARKERS | Expressed in brain cells | 35 | 0.28 | 2.00 | 0.00 | 1.00 | 1.00 |
| SMALL_GTPASE_MEDIATED_SIGNAL_TRANSDUCTION | Cell signalling | 48 | 0.24 | 1.99 | 0.01 | 1.00 | 1.00 |
| GSE17721_CPG_VS_GARDIQUIMOD_12H_BMDM_DN | Immune response | 98 | 0.17 | 1.98 | 0.00 | 1.00 | 1.00 |
| SLEBOS_HEAD_AND_NECK_CANCER_WITH_HPV_UP | Viral/immune response | 41 | 0.26 | 1.95 | 0.01 | 1.00 | 1.00 |
| LEIN_CHOROID_PLEXUS_MARKERS | Expressed in brain | 68 | 0.19 | 1.92 | 0.01 | 1.00 | 1.00 |
| GSE26928_EFF_MEM_VS_CENTR_MEM_CD4_TCELL_UP | Immune regulation | 105 | 0.16 | 1.91 | 0.01 | 1.00 | 1.00 |
| V$IK2_01 | Regulated by unknown transcription factor | 144 | 0.14 | 1.90 | 0.01 | 1.00 | 1.00 |
| PURINE_NUCLEOTIDE_BINDING | DNA replication/repair | 114 | 0.15 | 1.90 | 0.01 | 1.00 | 1.00 |
| YOKOE_CANCER_TESTIS_ANTIGENS | Cancer-related genes | 19 | 0.37 | 1.89 | 0.01 | 1.00 | 1.00 |
| NUCLEOTIDE_BINDING | DNA replication/repair | 122 | 0.15 | 1.89 | 0.01 | 1.00 | 1.00 |
| MODULE_323 | Brain immune regulation | 35 | 0.27 | 1.89 | 0.01 | 1.00 | 1.00 |
|  |  |  |  |  |  |  |  |
|  |  |  |  |  |  |  |  |
|  |  |  |  |  |  |  |  |
|  |  |  |  |  |  |  |  |
| **All glioma negative enrichment** |  |  |  |  |  |  |  |
| **GSE17721_LPS_VS_GARDIQUIMOD_6H_BMDM_UP** | **Viral/immune response** | **112** | **-0.22** | **-2.78** | **0.00** | **0.08** | **0.07** |
| REACTOME_RESPONSE_TO_ELEVATED_PLATELET_CYTOSOLIC_CA2_ | Clotting regulation | 46 | -0.31 | -2.53 | 0.00 | 0.28 | 0.40 |
| **GSE360_CTRL_VS_B_MALAYI_HIGH_DOSE_DC_UP** | **Parasite/immune response** | **103** | **-0.21** | **-2.49** | **0.00** | **0.25** | **0.48** |
| **ALCALAY_AML_BY_NPM1_LOCALIZATION_DN** | **Stem-cell renewal** | **101** | **-0.22** | **-2.49** | **0.00** | **0.19** | **0.50** |
| **GSE3982_MEMORY_CD4_TCELL_VS_BCELL_UP** | **Immune regulation** | **110** | **-0.20** | **-2.47** | **0.00** | **0.19** | **0.57** |
| KERLEY_RESPONSE_TO_CISPLATIN_UP | Response to cancer treatment | 24 | -0.41 | -2.36 | 0.00 | 0.33 | 0.84 |
| UNFOLDED_PROTEIN_BINDING | Stress response | 23 | -0.40 | -2.31 | 0.00 | 0.41 | 0.93 |
| SARRIO_EPITHELIAL_MESENCHYMAL_TRANSITION_UP | Invasiveness in breast cancer | 82 | -0.21 | -2.27 | 0.00 | 0.46 | 0.97 |
| GSE37416_12H_VS_24H_F_TULARENSIS_LVS_NEUTROPHIL_UP | Bacterial/immune response | 113 | -0.18 | -2.24 | 0.00 | 0.53 | 0.99 |
| OUILLETTE_CLL_13Q14_DELETION_UP | CLL-related genes | 39 | -0.30 | -2.23 | 0.00 | 0.48 | 0.99 |
| METALLOENDOPEPTIDASE_ACTIVITY | Essential enzyme activity | 18 | -0.45 | -2.23 | 0.00 | 0.46 | 0.99 |
| REACTOME_GAB1_SIGNALOSOME | Cell-growth signalling | 20 | -0.41 | -2.19 | 0.00 | 0.54 | 1.00 |
| CALCIUM_ION_BINDING | Cell signalling | 66 | -0.22 | -2.18 | 0.00 | 0.52 | 1.00 |
| BASAKI_YBX1_TARGETS_DN | Ovarian cancer-related genes | 199 | -0.13 | -2.15 | 0.00 | 0.58 | 1.00 |
| PID_P75NTRPATHWAY | Neuronal survival/differentiation | 35 | -0.30 | -2.14 | 0.00 | 0.58 | 1.00 |
| WANG_CLASSIC_ADIPOGENIC_TARGETS_OF_PPARG | Fat-cell differentiation | 17 | -0.44 | -2.13 | 0.00 | 0.61 | 1.00 |
| FERREIRA_EWINGS_SARCOMA_UNSTABLE_VS_STABLE_UP | Ewing's sarcoma-related genes | 95 | -0.18 | -2.07 | 0.01 | 0.79 | 1.00 |
| NEGATIVE_REGULATION_OF_NUCLEOBASENUCLEOSIDENUCLEOTIDE_AND_NUCLEIC_ACID_METABOLIC_PROCESS | Nucleic acid metabolism | 107 | -0.17 | -2.06 | 0.01 | 0.82 | 1.00 |
| RODWELL_AGING_KIDNEY_UP | Ageing in kidney | 264 | -0.11 | -2.06 | 0.01 | 0.79 | 1.00 |
| MORF_EIF3S6 | Protein synthesis | 45 | -0.26 | -2.06 | 0.00 | 0.75 | 1.00 |
|  |  |  |  |  |  |  |  |
|  |  |  |  |  |  |  |  |
|  |  |  |  |  |  |  |  |
|  |  |  |  |  |  |  |  |
|  |  |  |  |  |  |  |  |
|  |  |  |  |  |  |  |  |
| **GBM positive enrichment** |  |  |  |  |  |  |  |
| **KEGG_AMINO_SUGAR_AND_NUCLEOTIDE_SUGAR_METABOLISM** | **Glycolysis, DNA repair** | **26** | **0.46** | **2.84** | **0.00** | **0.04** | **0.04** |
| **GAVIN_FOXP3_TARGETS_CLUSTER_P6** | **T-cell immune cell regulation** | **57** | **0.29** | **2.64** | **0.00** | **0.11** | **0.19** |
| MODULE_239 | Liver cancer-related genes | 65 | 0.25 | 2.34 | 0.00 | 0.68 | 0.88 |
| SHETH_LIVER_CANCER_VS_TXNIP_LOSS_PAM3 | Liver cancer-related genes | 33 | 0.33 | 2.26 | 0.00 | 0.91 | 0.97 |
| GSE22886_IL2_VS_IL15_STIM_NKCELL_DN | Immune response to viral infection | 107 | 0.18 | 2.20 | 0.00 | 1.00 | 1.00 |
| GSE12845_IGD_POS_BLOOD_VS_PRE_GC_TONSIL_BCELL_UP | Immune regulation | 100 | 0.18 | 2.16 | 0.00 | 1.00 | 1.00 |
| GSE17721_CPG_VS_GARDIQUIMOD_24H_BMDM_UP | Immune response to viral/bacterial infection | 123 | 0.17 | 2.16 | 0.00 | 1.00 | 1.00 |
| GSE36392_TYPE_2_MYELOID_VS_NEUTROPHIL_IL25_TREATED_LUNG_UP | Immune response - inflammation | 111 | 0.17 | 2.15 | 0.00 | 0.96 | 1.00 |
| RESPONSE_TO_LIGHT_STIMULUS | Response to light exposure (can be important in cancer e.g. phytochemotherapy) | 27 | 0.34 | 2.12 | 0.00 | 1.00 | 1.00 |
| GSE32423_CTRL_VS_IL4_MEMORY_CD8_TCELL_UP | Activation of immune response (adaptive immunity) | 92 | 0.19 | 2.11 | 0.00 | 0.97 | 1.00 |
| MODULE_345 | Immune response | 71 | 0.21 | 2.11 | 0.01 | 0.90 | 1.00 |
| MORF_PCNA | DNA replication/repair | 28 | 0.33 | 2.10 | 0.00 | 0.86 | 1.00 |
| YANG_BREAST_CANCER_ESR1_BULK_UP | Hormone resistant breast cancer (affects cell cycle, apoptosis, DNA repair) | 15 | 0.46 | 2.10 | 0.00 | 0.80 | 1.00 |
| KEGG_BASAL_TRANSCRIPTION_FACTORS | Gene expression regulation | 19 | 0.41 | 2.10 | 0.00 | 0.75 | 1.00 |
| TIEN_INTESTINE_PROBIOTICS_6HR_DN | Immune response to bacteria | 90 | 0.18 | 2.08 | 0.01 | 0.80 | 1.00 |
| ZHENG_RESPONSE_TO_ARSENITE_DN | DNA damage response/DNA repair | 15 | 0.43 | 2.08 | 0.00 | 0.76 | 1.00 |
| WCAANNNYCAG_UNKNOWN | Regulated by unknown transcription factor | 127 | 0.16 | 2.07 | 0.00 | 0.73 | 1.00 |
| GSE17721_POLYIC_VS_PAM3CSK4_24H_BMDM_UP | Anti-viral immune response | 116 | 0.16 | 2.05 | 0.01 | 0.79 | 1.00 |
| SCHRAETS_MLL_TARGETS_UP | Epigenetic regulation | 18 | 0.40 | 2.05 | 0.00 | 0.77 | 1.00 |
| MODULE_206 | Breast cancer-related genes | 82 | 0.20 | 2.04 | 0.00 | 0.77 | 1.00 |
|  |  |  |  |  |  |  |  |
|  |  |  |  |  |  |  |  |
|  |  |  |  |  |  |  |  |
|  |  |  |  |  |  |  |  |
|  |  |  |  |  |  |  |  |
| **GBM negative enrichment** |  |  |  |  |  |  |  |
| GAZDA_DIAMOND_BLACKFAN_ANEMIA_PROGENITOR_DN | Tumour supressor/Ras oncogene/translational control | 29 | -0.36 | -2.38 | 0.00 | 1.00 | 0.80 |
| GENERATION_OF_PRECURSOR_METABOLITES_AND_ENERGY | Metabolism | 70 | -0.24 | -2.32 | 0.00 | 1.00 | 0.91 |
| BROWNE_HCMV_INFECTION_30MIN_DN | Response to viral infection | 83 | -0.21 | -2.32 | 0.00 | 0.82 | 0.91 |
| HOLLEMAN_VINCRISTINE_RESISTANCE_ALL_UP | Cell division/growth | 20 | -0.43 | -2.30 | 0.00 | 0.68 | 0.94 |
| CHR8Q13 | Genes in 8q13 | 23 | -0.38 | -2.16 | 0.00 | 1.00 | 1.00 |
| PID_NOTCH_PATHWAY | Notch signalling (proliferation/differentiation | 34 | -0.30 | -2.09 | 0.00 | 1.00 | 1.00 |
| GSE11864_UNTREATED_VS_CSF1_IFNG_IN_MAC_UP | Immune activation | 92 | -0.18 | -2.06 | 0.01 | 1.00 | 1.00 |
| PID_ATF2_PATHWAY | Stress response/apoptosis | 30 | -0.31 | -2.05 | 0.00 | 1.00 | 1.00 |
| SWEET_KRAS_ONCOGENIC_SIGNATURE | K-Ras oncogenic signalling | 38 | -0.28 | -2.05 | 0.01 | 1.00 | 1.00 |
| PEROXISOME | Metabolism | 31 | -0.31 | -2.04 | 0.00 | 1.00 | 1.00 |
| RHODOPSIN_LIKE_RECEPTOR_ACTIVITY | Signal transduction | 72 | -0.20 | -2.04 | 0.00 | 1.00 | 1.00 |
| MICROBODY | Metabolism | 31 | -0.31 | -2.04 | 0.01 | 1.00 | 1.00 |
| GSE24142_EARLY_THYMIC_PROGENITOR_VS_DN3_THYMOCYTE_ADULT_DN | T cell differentiation | 92 | -0.18 | -2.02 | 0.00 | 1.00 | 1.00 |
| PID_MYC_ACTIVPATHWAY | Targets of oncogene c-Myc (proliferation/differentiation) | 46 | -0.25 | -2.02 | 0.00 | 1.00 | 1.00 |
| FIGUEROA_AML_METHYLATION_CLUSTER_3_UP | AML-related genes | 84 | -0.19 | -2.02 | 0.00 | 1.00 | 1.00 |
| YOSHIOKA_LIVER_CANCER_EARLY_RECURRENCE_DN | Liver cancer recurrence | 36 | -0.28 | -2.02 | 0.01 | 1.00 | 1.00 |
| POTASSIUM_ION_TRANSPORT | Neuro-transmission | 32 | -0.30 | -2.01 | 0.01 | 1.00 | 1.00 |
| KEGG_FC_GAMMA_R_MEDIATED_PHAGOCYTOSIS | Phagocytosis | 53 | -0.23 | -2.01 | 0.00 | 1.00 | 1.00 |
| YAO_TEMPORAL_RESPONSE_TO_PROGESTERONE_CLUSTER_1 | Progesterone signalling | 42 | -0.26 | -2.00 | 0.01 | 0.98 | 1.00 |
|  |  |  |  |  |  |  |  |
|  |  |  |  |  |  |  |  |
|  |  |  |  |  |  |  |  |
|  |  |  |  |  |  |  |  |
| **Non-GBM positive enrichment** |  |  |  |  |  |  |  |
| **V$HEN1_01** | **Cell growth, development** | **99** | **0.23** | **2.66** | **0.00** | **0.17** | **0.17** |
| CLASPER_LYMPHATIC_VESSELS_DURING_METASTASIS_DN | Downregulated in metastasis | 19 | 0.43 | 2.27 | 0.00 | 1.00 | 0.98 |
| CHR16Q23 | genes in 16q23, commonly lost in gliomas | 16 | 0.47 | 2.24 | 0.00 | 1.00 | 0.99 |
| ERB2_UP.V1_UP | estrogen signalling - gene regulation | 112 | 0.17 | 2.14 | 0.00 | 1.00 | 1.00 |
| CHESLER_BRAIN_HIGHEST_GENETIC_VARIANCE | Expressed in brain | 18 | 0.41 | 2.13 | 0.00 | 1.00 | 1.00 |
| GSE17974_0H_VS_1H_IN_VITRO_ACT_CD4_TCELL_UP | T cell differentiation | 87 | 0.19 | 2.10 | 0.00 | 1.00 | 1.00 |
| GSE13485_DAY3_VS_DAY7_YF17D_VACCINE_PBMC_UP | Immune response to yellow fever virus | 76 | 0.20 | 2.07 | 0.01 | 1.00 | 1.00 |
| PEDRIOLI_MIR31_TARGETS_UP | Tumour supressor | 108 | 0.17 | 2.06 | 0.00 | 1.00 | 1.00 |
| GSE37416_0H_VS_3H_F_TULARENSIS_LVS_NEUTROPHIL_DN | Immune response to bacterial infection | 114 | 0.16 | 2.02 | 0.01 | 1.00 | 1.00 |
| ICHIBA_GRAFT_VERSUS_HOST_DISEASE_35D_DN | Immune regulation | 30 | 0.31 | 2.00 | 0.00 | 1.00 | 1.00 |
| GSE360_L_MAJOR_VS_B_MALAYI_LOW_DOSE_MAC_UP | Immune response to parasite | 97 | 0.17 | 1.99 | 0.00 | 1.00 | 1.00 |
| GSE32423_IL7_VS_IL4_MEMORY_CD8_TCELL_DN | Immune regulation | 99 | 0.17 | 1.99 | 0.01 | 1.00 | 1.00 |
| GSE25087_FETAL_VS_ADULT_TREG_DN | Immune regulation | 90 | 0.17 | 1.98 | 0.01 | 1.00 | 1.00 |
| MISSIAGLIA_REGULATED_BY_METHYLATION_UP | Regulated by methylation, response to DNA hypomethylating chemotherapy drug | 64 | 0.21 | 1.97 | 0.01 | 1.00 | 1.00 |
| LINDGREN_BLADDER_CANCER_CLUSTER_1_DN | downregulated in kidney cancer (e.g. candidate tumour suppressors) | 212 | 0.12 | 1.96 | 0.00 | 1.00 | 1.00 |
| GSE9037_WT_VS_IRAK4_KO_LPS_1H_STIM_BMDM_UP | Immune response to bacterial infection | 116 | 0.15 | 1.95 | 0.01 | 1.00 | 1.00 |
| GSE16522_ANTI_CD3CD28_STIM_VS_UNSTIM_MEMORY_CD8_TCELL_DN | Immune response | 114 | 0.15 | 1.93 | 0.00 | 1.00 | 1.00 |
| V$TFIIA_Q6 | Transcriptional control | 128 | 0.14 | 1.90 | 0.01 | 1.00 | 1.00 |
| CHR12P11 | Genes in 12p11, loss previously observed in gliomas | 18 | 0.37 | 1.90 | 0.01 | 1.00 | 1.00 |
| REACTOME_ABC_FAMILY_PROTEINS_MEDIATED_TRANSPORT | DNA repair/tumour therapy resitance/translation | 20 | 0.35 | 1.90 | 0.00 | 1.00 | 1.00 |
| **Non-GBM negative enrichment** |  |  |  |  |  |  |  |
| GSE17721_LPS_VS_POLYIC_24H_BMDM_DN | Immune response to viruses/bacteria | 107 | -0.22 | -2.54 | 0.00 | 0.48 | 0.36 |
| V$VDR_Q3 | Regulated by Vitamin D | 111 | -0.20 | -2.41 | 0.00 | 0.68 | 0.73 |
| JECHLINGER_EPITHELIAL_TO_MESENCHYMAL_TRANSITION_DN | Negative regulators of metastasis/invasiveness | 38 | -0.32 | -2.33 | 0.00 | 0.78 | 0.89 |
| GSE17721_LPS_VS_GARDIQUIMOD_6H_BMDM_UP | Immune response to viruses/bacteria | 115 | -0.19 | -2.30 | 0.00 | 0.76 | 0.95 |
| GSE24142_DN2_VS_DN3_THYMOCYTE_ADULT_UP | Immune regulation | 105 | -0.19 | -2.23 | 0.00 | 0.95 | 0.99 |
| GSE3982_EOSINOPHIL_VS_MAST_CELL_DN | Immune regulation | 102 | -0.18 | -2.11 | 0.00 | 1.00 | 1.00 |
| PARK_TRETINOIN_RESPONSE_AND_PML_RARA_FUSION | Retinoic acid signalling (differentiation, apoptosis) | 18 | -0.42 | -2.09 | 0.00 | 1.00 | 1.00 |
| GSE17721_LPS_VS_POLYIC_0.5H_BMDM_UP | Immune response to viruses/bacteria | 112 | -0.17 | -2.07 | 0.00 | 1.00 | 1.00 |
| GSE1448_ANTI_VALPHA2_VS_VBETA5_DP_THYMOCYTE_DN | Immune response | 106 | -0.17 | -2.05 | 0.00 | 1.00 | 1.00 |
| POTTI_5FU_SENSITIVITY | genes sensitive to 5-FU chemotherapy (blocks DNA replication) | 17 | -0.42 | -2.05 | 0.00 | 1.00 | 1.00 |
| KIM_WT1_TARGETS_8HR_DN | Regulated by WT1 (expressed in gliomas) | 71 | -0.21 | -2.05 | 0.01 | 1.00 | 1.00 |
| AGUIRRE_PANCREATIC_CANCER_COPY_NUMBER_DN | Pancreatic cancer-related genes | 127 | -0.15 | -2.04 | 0.01 | 1.00 | 1.00 |
| GSE17721_PAM3CSK4_VS_GADIQUIMOD_8H_BMDM_UP | immune response to viruses/bacteria | 105 | -0.17 | -2.03 | 0.00 | 1.00 | 1.00 |
| ALCALAY_AML_BY_NPM1_LOCALIZATION_DN | Stem-cell renewal | 100 | -0.17 | -2.02 | 0.00 | 1.00 | 1.00 |
| AIYAR_COBRA1_TARGETS_DN | DNA repair | 20 | -0.38 | -2.00 | 0.00 | 1.00 | 1.00 |
| GSE37416_CTRL_VS_6H_F_TULARENSIS_LVS_NEUTROPHIL_DN | Bacterial immune response | 93 | -0.18 | -2.00 | 0.00 | 1.00 | 1.00 |
| WATANABE_RECTAL_CANCER_RADIOTHERAPY_RESPONSIVE_UP | Response to radiotherapy | 56 | -0.22 | -2.00 | 0.00 | 1.00 | 1.00 |
| BOYAULT_LIVER_CANCER_SUBCLASS_G123_UP | Liver cancer-related genes | 24 | -0.34 | -1.99 | 0.01 | 1.00 | 1.00 |
| GSE1460_INTRATHYMIC_T_PROGENITOR_VS_DP_THYMOCYTE_DN | Immune regulation | 101 | -0.17 | -1.98 | 0.01 | 1.00 | 1.00 |
| EXCRETION | Metabolism | 23 | -0.34 | -1.97 | 0.00 | 1.00 | 1.00 |

**Supplementary Table 5: Gene-set enrichment analysis (GSEA) of glioma-associated genes.** GSEA was performed on gene-based *SKAT* *P* values generated for all glioma as well as GBM and non-GBM subtypes. “Positive enrichment” corresponds to gene sets enriched for positively associated (low *SKAT* *P* value) genes, “Negative enrichment” corresponds to gene sets enriched for negatively-associated (high *SKAT P* value) genes. Gene sets in bold are those passing advocated thresholds (*i.e.* *P_GSEA_* < 0.05, FDR *q* < 0.25). ES, effect size; NES, normalised effect size; FDR, false discovery rate; FWER, family-wise error rate.
