# Supplementary material for: Search for new loci and low-frequency variants influencing glioma risk by exome-array analysis
Source: Eur J Hum Genet. 2015 Aug 12;24(5):717–24. doi: 10.1038/ejhg.2015.170 (PMC4677454; doi:10.1038/ejhg.2015.170)
Supplement: Supplementary Table 6 [file ejhg2015170x10.docx]

| **Minor allele count** | **Total missense variants** | **Exome array missense variants (% of total)** |
| --- | --- | --- |
| 0+ | 97,898 | 62,569 (64%) |
| 1+ | 79,652 | 57,113 (72%) |
| 2+ | 69,088 | 52,148 (75%) |
| 3+ | 62,475 | 48,189 (77%) |
| 4+ | 57,867 | 45,074 (78%) |
| 5+ | 54,463 | 42,569 (78%) |
| 10+ | 44,645 | 34,853 (78%) |
| 50+ | 27,781 | 21,248 (76%) |
| 100+ | 22,568 | 17,095 (76%) |
| 500+ | 13,457 | 10,082 (75%) |
| 1,000+ | 9,739 | 7,298 (75%) |
| 2,000+ | 5,313 | 3,943 (74%) |

**Supplementary Table 6: Frequency distribution of missense variants in 3,781 UK10K genomes.** Total missense variants for given frequency are shown along with the total numbers present on the exome array.
